# Biological Activities and Phytochemical Profiles of Extracts from Different Parts of Bamboo (*Phyllostachys pubescens*)

**DOI:** 10.3390/molecules19068238

**Published:** 2014-06-18

**Authors:** Akinobu Tanaka, Qinchang Zhu, Hui Tan, Hiroki Horiba, Koichiro Ohnuki, Yasuhiro Mori, Ryoko Yamauchi, Hiroya Ishikawa, Akira Iwamoto, Hiroharu Kawahara, Kuniyoshi Shimizu

**Affiliations:** 1 Department of Agro-environmental Sciences, Faculty of Agriculture, Kyushu University, 6-10-1 Hakozaki, Higashi-ku, Fukuoka, 812-8581, Japan; E-Mails: akinobu0917@gmail.com (A.T.); zhu_qinchang@hotmail.com (Q.Z.); thth229@agr.kyushu-u.ac.jp (H.T.); h2.horiba0728@agr.kyushu-u.ac.jp (H.H.); 2 Department of Biological and Environmental Chemistry, Kinki University, Kayanomori 11-6, Iizuka, Fukuoka, 820-8555, Japan; E-Mail: ohnuki_usi@yahoo.co.jp; 3 Fukuoka Prefecture Institute of Agricultural and Forest Resources, 1438-2, Toyota, Yamamoto-town, Kurume, Fukuoka, 839-0827, Japan; E-Mail: mori-y9360@ffrec.pref.fukuoka.jp; 4 International College of Arts and Sciences, Fukuoka Women’s University, Fukuoka 813-8529, Japan; E-Mails: yamauchi-r@fwu.ac.jp (R.Y.); ishikawa@fwu.ac.jp (H.I.); 5 Department of Materials Science and Chemical Engineering, Kitakyushu National College of Technology, 5-20-1 Shii, Kokuraminami-ku, Kitakyushu, Fukuoka, 802-0985, Japan; E-Mails: iwamoto.akira.344@s.kyushu-u.ac.jp (A.I.); hk128@kct.ac.jp (H.K.)

**Keywords:** *Phyllostachys pubescens*, cosmetics, anti-melanogenesis, antioxidation, antibacterial, anti-allergy, HPLC, LC-MS-IT-TOF

## Abstract

Besides being a useful building material, bamboo also is a potential source of bioactive substances. Although some studies have been performed to examine its use in terms of the biological activity, only certain parts of bamboo, especially the leaves or shoots, have been studied. Comprehensive and comparative studies among different parts of bamboo would contribute to a better understanding and application of this knowledge. In this study, the biological activities of ethanol and water extracts from the leaves, branches, outer culm, inner culm, knots, rhizomes and roots of *Phyllostachys pubescens*, the major species of bamboo in Japan, were comparatively evaluated. The phytochemical profiles of these extracts were tentatively determined by liquid chromatography-mass spectrometry (LC-MS) analysis. The results showed that extracts from different parts of bamboo had different chemical compositions and different antioxidative, antibacterial and antiallergic activities, as well as on on melanin biosynthesis. Outer culm and inner culm were found to be the most important sources of active compounds. 8-*C*-Glucosylapigenin, luteolin derivatives and chlorogenic acid were the most probable compounds responsible for the anti-allergy activity of these bamboo extracts. Our study suggests the potential use of bamboo as a functional ingredient in cosmetics or other health-related products.

## 1. Introduction

Bamboo is well known for its extensive use. Besides being used in building construction, its roots and leaves have been used medicinally. Studies have revealed that bamboo leaves have antioxidant, anticancer and antibiotic properties [1,2]. In previous studies, various active compounds, such as flavones, glycosides, phenolic acids, coumarin lactones, anthraquinones and amino acids, have been isolated from the leaves [3,4,5,6,7]. 2, 6-Dimethoxy-*p*-benzoquinone isolated from the skin of bamboo trees and two chitin-binding peptides (Pp-AMP1 and Pp AMP2) isolated from bamboo shoots were found to have antibiotic activities [8,9]. Stigmasterol and dihydrobrassicasterol isolated from the skin of bamboo shoot showed antibacterial activity [10], as well as tricin and taxifolin [11].

**Figure 1:**
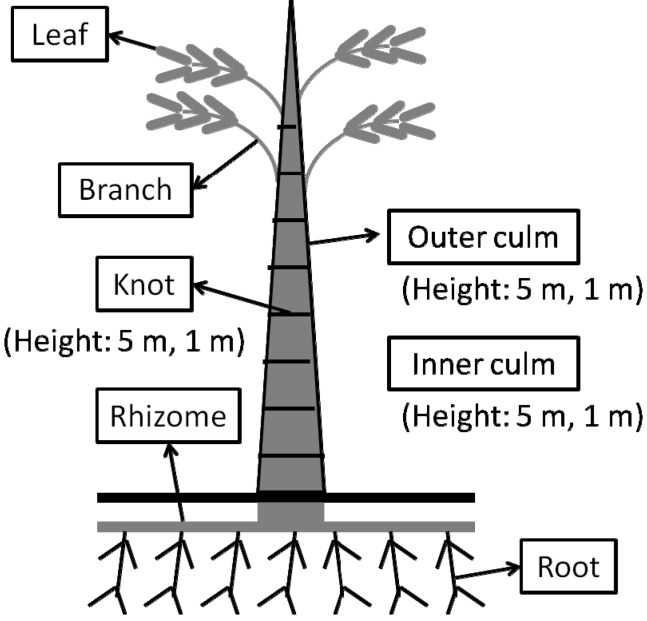
Parts of *P. pubescens* used in present study. *P. pubescens* plants were separated into leaves, branches, outer culm, inner culm, knots, rhizomes and roots. Outer culm, inner culm and knot samples were obtained separately at the height of 5.0 ± 0.3 and 1.0 ± 0.3 meter above ground level.

*Phyllostachys pubescens (P. pubescens)* is the major species of bamboo in Japan, which is widely distributed through the country. In fact, how to stop its further spread is a problem in Japan [12]. Hence, new applications of bamboo in various commercial industries are being explored. Several studies have been performed to demonstrate the use of *P. pubescens* in terms of its biological activity. These studies have mainly focused on extracts from specific parts of *P. pubescens*. For example, the antioxidant activity of leaves [13] and shoots [14], antiallegic [15] and anticancer [16] activities of leaves and branches and antibacterial activities of stems [8], shoots [9], and shoot skins [17]. However, comprehensive and comparative studies of extracts from all parts of bamboo using the same extraction solvent have not been done. In this study, *P. pubescens* was separated into 10 parts, including leaves, branches, outer culm (5 m and 1 m above the ground, respectively), inner culm (5 m and 1 m above the ground, respectively), knots (5 m and 1 m above the ground, respectively), rhizomes and roots (Figure 1). These parts of bamboo were extracted by ethanol and hot water. All resulting extracts were subjected to four assays for bioactivities that are usually of interest to the cosmetics industry. They are melanin synthesis assay, antioxidant assay, antibacterial assay and antiallergic assay. At the same time, the chromatographic profiles of these extracts were determined and their components were partially identified using liquid chromatography-mass spectrometry (LC-MS). Through these tests and analysis, the potential use of bamboo in health-related industries, especially in cosmetics industry was evaluated. 

## 2. Results and Discussion

In the present study, the ethanol and hot water extracts of various parts of *P. pubescens* (Figure 1) were examined for several biological activities suing the melanin biosynthesis assay (Table 1), antioxidant assay (Table 2), antibacterial assay (Table 3) and immunoglobulin E (IgE) production assay (Table 4). Their phytochemical profiles were also investigated through LC-MS analysis (Table 5, Table 6 and Figures S1–S3). 

### 2.1. Activity on Melanin Biosynthesis

Table 1 shows the effect of the ethanol extracts and the hot water extracts of *P. pubescens* on melanin biosynthesis and cell proliferation of B16 melanoma cells. After treating with different concentrations of the extract for 3 days, B16 melanoma cells were examined for cell viability (CV) and melanin content (MC). The cell viability was measured by the classic MTT assay, while the melanin content was determined by the absorbance at 405 nm. One important concept when selecting bioactive extracts that modulate skin pigmentation for cosmetics is that they should have minimal effects on cell proliferation and/or survival. 

As shown in Table 1, the ethanol extracts of branches, and outer culm (5 m, 1 m) showed melanin biosynthesis inhibitory activity (Type A) in a dose-dependent manner. The ethanol extract of branches inhibited biosynthesis of melanin at 120 μg/mL (CV was 86.3% and MC was 56.0%). The ethanol extract of the outer culm at 5 m showed activity at 120 and 60 μg/mL (CVs were 86.9% and 98.9%; MCs were 44.4 and 72.2%, respectively). The ethanol extract of the outer culm at 1 m showed activity at 120 and 60 μg/mL (CVs were 124 and 109%; MCs were 49.5 and 79.8%, respectively). On the other hand, the ethanol extracts of the inner culm at both heights (5 m, 1 m), knots at 1 m, rhizomes and roots showed selective melanin biosynthesis-stimulating activity (Type B). The ethanol extract of the inner culm at 5 m stimulated biosynthesis of melanin at 60 μg/mL (CV was 88.5% and MC was 109%). Also, the ethanol extract of the inner culm at 1 m stimulated biosynthesis of melanin at 120, 60 and 20 μg/mL (CVs were 106%, 106% and 103%; MCs were 142%, 151% and 134%, respectively). The ethanol extract of knots at 1 m showed activity at 120 and 60 μg/mL (CVs were 100% and 97.0%; MCs were 133% and 119%, respectively). 

Table 1Effect of the (**a**) ethanol extracts and (**b**) the hot water extracts of *P. pubescens* on melanin biosynthesis and cell proliferation of B16 melanoma cells.

**Table:** (**a**)

Part	Ethanol extract								
	120 μg/mL			60 μg/mL			20 μg/mL		
	CV	MC	Type	CV	MC	Type	CV	MC	Type
Leaf	91.2 ± 1.06	105 ± 5.52	-	96.7 ± 8.10	99.0 ± 8.53	-	101 ± 1.98	103 ± 1.32	-
Branch	86.3 ± 1.71	56.0 ± 7.90	A,C	91.0 ± 2.74	75.6 ± 3.52	-	90.2 ± 0.84	84.6 ± 2.09	-
Outer culm (5 m)	86.9 ± 10.1	44.4 ± 5.64	A,C	98.9 ± 0.98	72.2 ± 1.55	A	112.9 ± 4.27	104 ± 2.34	-
Outer culm (1 m)	124 ± 9.08	49.5 ± 5.38	A	109 ± 1.20	79.8 ± 3.19	A	124 ± 9.08	112 ± 2.97	-
Inner culm (5 m)	98.8 ± 2.09	110 ± 2.67	-	88.5 ± 10.5	109 ± 9.68	B,C	98.0 ± 1.09	106 ± 8.01	-
Inner culm (1 m)	106 ± 1.80	142 ± 2.87	B	106 ± 5.63	151.9 ± 9.59	B	103 ± 3.32	134 ± 3.59	B
Knot (5 m)	93.6 ± 5.51	101 ± 9.90	-	90.5 ± 1.96	104 ± 0.69	-	93.4 ± 4.86	101 ± 3.27	-
Knot (1 m)	100 ± 6.22	133 ± 20.0	B	97.0 ± 7.14	119 ± 2.97	B	96.8 ± 6.46	107 ± 6.67	-
Rhizome	120 ± 2.97	137 ± 19.0	-	118 ± 1.88	144 ± 21.6	B	114 ± 4.44	121 ± 4.2	-
Root	91.5 ± 2.68	118 ± 6.78	B	88.6 ± 1.2	126 ± 5.53	B, C	104 ± 10.1	111 ± 12.7	-

**Table:** (**b**)

Part	Hot water extract								
	120 μg/mL			60 μg/mL			20 μg/mL		
	CV	MC	Type	CV	MC	Type	CV	MC	Type
Leaf	109 ± 9.06	114 ± 7.71	-	107 ± 8.35	108 ± 11.1	-	109 ± 9.06	143.8 ± 2.22	B
Branch	79.7 ± 10.7	84.8 ± 8.16	C	77.3 ± 3.67	87.2 ± 1.39	C	83.1 ± 7.42	109 ± 2.73	B,C
Outer culm (5 m)	121 ± 6.83	78.9 ± 6.10	A	125 ± 3.76	93.3 ± 13.0	A	138 ± 2.22	94.6 ± 6.76	A
Outer culm (1 m)	118 ± 2.81	125 ± 16.5	-	114 ± 2.99	104 ± 16.5	-	116 ± 6.16	117 ± 14.8	-
Inner culm (5 m)	97.5 ± 7.10	104 ± 6.62	-	114 ± 9.85	105 ± 15.2	-	96.2 ± 9.62	101 ± 2.82	-
Inner culm (1 m)	120 ± 5.10	113 ± 17.6	-	110 ± 15.0	108 ± 0.28	-	113 ± 0.76	102 ± 1.97	-
Knot (5 m)	79.1 ± 6.23	94.8 ± 5.69	C	85.8 ± 2.81	109 ± 9.61	B,C	77.7 ± 1.65	101 ± 1.86	B,C
Knot (1 m)	90.1 ± 12.9	99.5 ± 14.1	-	103 ± 17.5	103 ± 22.0	-	78.0 ± 8.30	96.8 ± 2.91	C
Rhizome	111 ± 3.12	88.9 ± 2.01	A	123 ± 9.64	97.3 ± 15.3	A	115 ± 7.03	93.2 ± 12.6	A
Root	99.9 ± 2.22	114 ± 10.4	-	94.3 ± 4.94	121 ± 9.18	B	98.2 ± 3.49	116 ± 11.2	B

Data presented as means ± SD (n = 3). CV, cell viability (%); MC, melanin content (%). Type A (CV-MC ≥ 20): melanin-biosynthesis-inhibitory activity; Type B (MC-CV ≥ 20): melanin-biosynthesis-stimulating activity; Type C (CV ≤ 90%): cytotoxicity. Arbutin (100μg/mL) was used as the positive control for melanin-biosynthesis inhibition. Its CV and MC were 94.7% and 46.5%, respectively. It belongs to the Type A.

It is notable that ethanol extracts of knots from 1 m but not from 5 m showed activity. Also, ethanol extracts of rhizomes (60 μg/mL) and roots (120 and 60 μg/mL) showed melanin-biosynthesis-stimulating activity (CV was 118% and MC was 144% for rhizomes; CVs were 91.5% and 88.6% and MCs were 118% and 126% for roots, respectively). In this assay, DMSO was used to dissolve ethanol extracts, and its final concentration was 0.2%. Under such concentration, DMSO didn’t show cytotoxicity to the B16 melanoma cells (MTT assay, data not shown). Because the results were calculated basing on the comparison with DMSO-treated group, DMSO used in this assay should not affect the results.

**Table 2:** Antioxidant activity of the ethanol extracts and hot water extracts from *P. pubescens*.

Part	Ethanol Extract			Hot Water Extract		
	ORAC (mgTE/mg)	SOD Unit (U/μg)	ABTS IC_50_ (μg/mL)	ORAC (mgTE/mg)	SOD Unit (U/μg)	ABTS IC_50_ (μg/mL)
Leaf	0.07 ± 0.02	nd	nd	0.37 ± 0.08	nd	306.7 ± 5.7
Branch	0.69 ± 0.04	4.4 ± 1.0	350.6 ± 7.1	0.84 ± 0.01	0.6 ± 0.0	179.5 ± 3.6
Outer culm (5 m)	0.52 ± 0.07	0.2 ± 0.0	nd	0.65 ± 0.03	1.0 ± 0.3	113.7 ± 18.2
Outer culm (1 m)	0.18 ± 0.01	0.1 ± 0.0	nd	0.59 ± 0.05	0.8 ± 0.1	140.1 ± 1.4
Inner culm (5 m)	0.72 ± 0.09	0.9 ± 0.1	88.5 ± 0.8	0.29 ± 0.03	nd	198.3 ± 3.0
Inner culm (1 m)	1.35 ± 0.14	0.2 ± 0.0	373.8 ± 3.2	0.30 ± 0.00	nd	231.9 ± 4.9
Knot (5 m)	0.22 ± 0.00	nd	nd	0.29 ± 0.02	nd	245.0 ± 4.2
Knot (1 m)	0.22 ± 0.00	nd	nd	0.28 ± 0.01	nd	240.7 ± 1.9
Rhizome	0.71 ± 0.02	0.1 ± 0.0	171.5 ± 5.4	0.31 ± 0.00	nd	266.7 ± 6.8
Root	0.05 ± 0.03	nd	nd	0.54 ± 0.02	0.2 ± 0.0	209.7 ± 7.8

Data presented as means ± SD (n = 3); ORAC, oxygen radical absorbance capacity; SOD, superoxide dismutase; ABTS, 2,2'-azino-bis (3-ethylbenzothiazoline-6-sulphonic acid); ORAC values are expressed as relative Trolox equivalents per milligram; nd, not determined because the value is below the detection limit.

**Table 3:** Antibacterial activity of the ethanol extracts and the hot water extracts of *P. pubescens*.

Part	Ethanol Extract			Hot Water Extract		
	Growth Inhibition		MIC/MBC (μg/mL)	Growth Inhibition		MIC/MBC (μg/mL)
	Concentration (μg/mL)	Rate (% *vs*. Control) *		Concentration (μg/mL)	Rate (% *vs*. Control) *	
Leaf	600	-	-	600	98.1 ± 0.47	1200/1600
Branch	1200	-	-	1200	97.6 ± 1.61	1400/>1400
Outer culm (5 m)	600	97.8 ± 11.6	400/1600	600	13.7 ± 6.89	nd
Outer culm (1 m)	600	100 ± 0.47	400/1600	600	12.1 ± 9.30	nd
Inner culm (5 m)	600	-	-	600	99.5 ± 1.68	>1600
Inner culm (1 m)	600	-	-	600	-	-
Knot (5 m)	600	-	-	600	31.2 ± 15.0	nd
Knot (1 m)	600	-	-	600	-	-
Rhizome	1200	-	-	1200	44.1 ± 12.9	nd
Root	1200	-	-	1200	52.4 ± 15.7	nd

* Data presented as means ± SD (n = 3). MIC: minimum inhibitory concentration; MBC: minimum bactericidal concentration. -: no antibacterial activity; nd: non-detect. Sorbic acid (200μg/mL) was used as positive control and its inhibition rate was 73.7% ± 10.7%.

**Table 4:** Anti-allergy activity of the ethanol extracts and the hot water extracts of *P. pubescens*.

Part	IgE Production (%)	
	Ethanol Extract	Hot Water Extract
Leaf	97.3 ± 38.9	57.2 ± 9.28 **
Branch	227 ± 95.8	103 ± 45.4
Outer culm (5 m)	144 ± 27.7	70.7 ± 13.1 *
Outer culm (1 m)	137 ± 109	64.1 ± 6.47 **
Inner culm (5 m)	110 ± 39.6	64.1 ± 18.1 *
Inner culm (1 m)	93.1 ± 15.0	66.9 ± 19.8
Knot (5 m)	115 ± 61.9	64.1 ± 10.3 **
Knot (1 m)	107 ± 39.8	73.8 ± 14.8
Rhizome	60.6 ± 29.8	62.8 ± 15.4 *
Root	75.1 ± 31.0	73.7 ± 19.4

Date presented as means ± SD (n = 3). Concentration of each sample is 60 μg/mL. Significant differences between control and each extract were determined by Student’s t-test: * *p* < 0.05, ** *p* < 0.01.

The hot water extracts of the outer culm (5 m) and rhizomes showed melanin-biosynthesis-inhibitory activity (Type A behavior) at 120, 60 and 20 μg/mL (CVs were 121%, 125% and 138% and MCs were 78.9, 93.3 and 94.6%, respectively for outer culm at 5 m; CVs were 111%, 123% and 115%, and MCs were 88.9%, 97.3% and 93.2%, for rhizomes). On the other hand, the hot water extracts of leaves, branches, knots at 5 m, and roots showed melanin-biosynthesis-stimulating activity (Type B behavior). The hot water extract of leaves showed activity at 20 μg/mL (CV was 109% and MC was 143%). The hot water extract of branches showed activity at 20 μg/mL (CV was 83.1% and MC was 109%). The latter extract showed relatively strong cytotoxicity at tested concentrations and was classified as type C. The hot water extract of knots at 5 m showed activity at 60 and 20 μg/mL (CVs were 85.8% and 77.7%; MCs were 109% and 101%, respectively). This extract also showed relatively strong cytotoxicity at tested concentrations and was classified as type C (CVs were 79.1%, 85.8% and 77.7%, respectively). The hot water extract of roots showed activity at 60 and 20 μg/mL (CVs were 94.3% and 98.2%; MCs were 121% and 116%, respectively). 

The melanin-biosynthesis-inhibition activity of extract prepared from bamboo indicates its potential use as a skin-whitening agent. On the other hand, melanin-biosynthesis-stimulating activity is important for skin tanning agent and hair dyes.

### 2.2. Antioxidant Activity

Table 2 shows the antioxidant activity of the ethanol extracts and the hot water extracts of *P. pubescens*. The ethanol extract of the inner culm at 1 m showed the highest ORAC value (1.35 mgTE/mg) in all tested extracts. Other extracts showed ORAC values from 0.07 to 0.84 mgTE/mg. SOD-like activities were detected from several extracts. The ethanol extract of branches showed the strongest SOD-like activity (4.4 U/μg). Also, the ethanol extracts of the outer culm at both heights, inner culm at both heights, and rhizomes and the hot water extracts of branches, outer culm at both heights and roots showed SOD-like activities (0.1 – 1.0 U/μg). The ethanol extract of the inner culm at 5 m showed the strongest ABTS radical decolorization activity in all tested extracts (IC_50_ = 88.5 μg/mL). The IC_50_s could be calculated from all hot water extracts. However, among the ethanol extracts, only those of the branches, inner culm at both heights, and rhizomes showed enough activity to calculate their IC_50_s. The hot water extracts tended to show stronger activity than the ethanol extracts. Skin is a major potential target of oxidative stress. Oxidative stress enhances melanin biosynthesis, damages DNA, and may induce proliferation of melanocytes [18]. Therefore, antioxidants can reduce hyperpigmentation. Considering both the melanin-biosynthesis-inhibiting and antioxidant activities of bamboo extracts, they have potential as skin-whitening agents.

There was no correlation between the intensity of ORAC, SOD and ABTS. This is not a surprising result, because these three assays evaluate the activity throughout quite different mechanisms. The ORAC assay is based on hydrogen atom transfer reactions and the ABTS inhibition rates are based on the electron-transfer ability of the sample’s components. Also, SOD-like activity is based on the antioxidative enzyme-like activity of the sample’s components.

### 2.3. Antibacterial Activity

Antibacterial activity against *Staphylococcus aureus* is an important attribute of skin cosmetics, because the proliferation of bacteria causes skin problems such as acne, comedo, papules, cellulitis and allergies [19,20]. Therefore, we also evaluated the antibacterial activity of the extracts from *P. pubescens*. Table 3 shows the antibacterial activity of the ethanol extracts and the hot water extracts of *P. pubescens*. The ethanol extracts of the outer culm at both heights and the hot water extracts of leaves, branches and inner culm at 5 m almost completely inhibited the growth of bacteria (growth inhibition rates were 97.8, 100, 98.1, 97.6 and 99.5, respectively). For the part of outer culm at the height of both 5 m and 1 m, the ethanol extracts showed strong antibacterial activity (growth inhibition rates were 97.8% and 100% for 5 and 1 m, respectively), while the hot water extract didn’t show good activity (growth inhibition rates were 13.7% and 12.1%), suggesting that the antibacterial constituents in the outer culm are lipophilic The MIC (minimum inhibitory concentration) and MBC (minimum bactericidal concentration) of active extracts were further determined using higher concentrations. The ethanol extracts of outer culm at both heights of 5 m and 1 m showed the lowest MIC (400 μg/mL). Three extracts showed minimum bacterialcidal effect at 1,600 μg/mL. They are ethanol extracts of outer culm (5 m and 1 m) and hot water extract of leaf. For the hot water extracts that showed weaker activity, the MIC/MBC were not detected because of the low activity of them at the concentrations close to their maximum solubility. Most of hot water extracts showed antibacterial activity at various inhibition rates (12.1%–100%). However, among the ethanol extracts, only the outer culm at 5 and 1 m showed antibacterial activity. The hot water extracts tended to show stronger antibacterial activity than the ethanol extracts. The antibacterial activity of bamboo would be useful in keeping skin healthy.

### 2.4. Anti-Allergy Activity

Some components in cosmetics cause side effect of allergies, the addiction of ingredients with anti-allergy activity to the cosmetics will be helpful to avoid such side effect. 

**Table 5:** Partial characterization of ethanol extracts of various parts of bamboo by LCMS-IT-TOF.

Part	Comp. *	tR (min)	UV λ (nm)	MS		MS/MS	Tentative Identification
				[M+H]^+^	Main Fragments		
Leaf	1	7.79	254,326	595.1222	563.2069 385.1727 401.2092	472.1109 325.0804 457.1063 379.0754	Di-*C*,*C*-hexosyl apigenin
	2	9.75	254,326	583.2920	249.1044 331.6180 419.1789 532.1011	-	Tricin derivative
	3	10.99	254, 326	547.0304	214.9922 405.2008 316.5860 474.0304	391.0690 260.0637 419.1118	Not identified
	4	13.68	254,326	639.1865	561.3183 427.5450 357,1133 331.0914	331.0833	*O*-hexosyl-*O*- deoxyhexosyl tricin
	5	13.96	254,326	493.1227	235.0158 314.0666	331.0777	*O*-hexosyl tricin
Branch	6	11.01	254,326	433.1361	313.0428 214.9618	283.0601 337.0809 415.0739 162.9025	6-*C*-glucosyl apigenin (isovitexin)
Outer culm (5 m)	7	4.48	254	351.0937	196.9942 442.0793 253.0631 156.0012	269.3353 315.1813 211.5120 153.9859	Not identified
	1	7.55	254,326	595.2048	401.1621 563.2534 385.1795 511.1788	383.1592 373.1058 318.5544 244.3389	Di-*C*,*C*-hexosyl apigenin
	4	13.65	254,326	639.1805	561.3528, 589.1020 315.0292 173.9611	331.0775 270.0903 415.4247	*O*-hexosyl-*O*- deoxyhexosyl tricin
Outer culm (1 m)	1	7.70	254,326	595.1777	563.2181 385.2050 457.1007 214.9845	325.0885 427.1041 457.0921 379.0553	Di-*C*,*C*-hexosyl apigenin
	8	9.76	254,326	582.2133	249.1086 331.6027 403.1153	371.1527 249.0799	Tricin derivative
	9	12.34	254,326	549.1879	197.1128 384.5780 498.6190	447.1168 495.1613	Not identified
	4	13.57	254,326,	639.2403	561.3504 215.0037 289.1380 401.0721 485.6067	331.0808	*O*-hexosyl-*O*- deoxyhexosyl tricin
Inner culm (5 m)	1	7.71	254,326	595.1699	401.0721 215.0030 563.2233 379.1075	427.0928 457.1007 295.0754 379.0791	Di-*C*,*C*-hexosyl apigenin
Inner culm (1 m)	10	10.69	254	581.1780	401.1565 140.0316 214.9463 284.7471	305.0035 219.0867 131.0860	Not identified
	11	10.92	254,326	581.2469,	215.0359 256.0465 329.6107 155.9239	173.9575	Not identified
Knot (5 m)	12	7.77	254,326	597.1854	214.9895 197.0173 256.0255 433.8491	149.0515 165.7342 223.7458	Not identified
Knot (1 m)	2	9.81	254,326	583.1954	249.1038 401.1687 331.5832 237.1147	131.0739 232.1788 231.0688	Tricin derivative
	13	17.03	254,326	441.1956	354.2400 212.0524 154.9682	265.1592 177.0511	Not identified
Rhizome	14	15.16	254,326	323.1311	256.0713 181.0395 196.9698 215.0161 240.9718	169.2761	Not identified
	15	15.92	254,326	353.1676	181.0075 156.0012 255.9951 214.9742	177.0655 145.0326 337.1738	Not identified
	16	16.52	254,326	411.1811	215.0015 206.1046 266.0636 367.1154	235.1485 147.0246 265.1572 177.0882	Not identified
	13	17.01	254, 326	441.2149	289.0373 197.0155 154.0150 255.9951	265.1526 177.0535 145.0496	Not identified
	17	18.43	254,326	455.2147	181.0407 214.9568 197.0268 381.6146 266.0714 308.6563	173.9925 124.2863 249.5310	Not identified
	18	22.73	254,326	445.1614	214.9897 181.0442 197.0006 317.1962 405.2211 283.1105	427.1142 177.9142 362.6228 114.6710	Not identified
Root	1	7.70	254,326	595.1482	196.9985 498.0875 542.1194 325.0225 249.1168	409.1006 457.1415 369.0885 421.0878 439.1011	Di-*C*,*C*-hexosyl apigenin
	2	9.90	254,326	583.2460	249.1353 331.5924 605.1899 360.0969 214.9603 281.0794 403.5934	207.3230 520.5871 286.0984 412.9828 388.1342	Not identified
	19	11.05	245,326	579.1288	214.9826 247.0254 350.0942 164.0768	411.1128 429.1186 393.1004 349.0813 409.1005 295.0939	Not identified
	20	12.37	245,326	549.1083	531.1381 197.0006 457.1618 337.1038 382.8247 139.9865	531.1381 197.0006 457.1618 337.1038 382.8247 139.9865	Di-*C*-glycosyl apigenin
	5	13.92	245,326	493.1157	295.0853 338.5214 197.0107 475.3166	331.0791 442.3596 244.4431	*O*-hexosyl tricin

* Compounds that show pseudomolecular ions in mass spectra in both positive and negative ion modes were listed here and indicated in corresponding chromatograms in Figure S1.

**Table 6:** Partial characterization of water extracts of different parts of bamboo by LCMS-IT-TOF.

Part	Comp. *	tR (min)	UV λ (nm)	MS		MS/MS	Tentative Identification
				[M+H]^+^	Main Fragments		
Leaf	21	8.36	254,278	346.0437	206.9890 235.0134 173.0079 242.0372	145.0514 292.1701 177.0293 313.7123	Not identified
	22	12.69	254,278	355.0828	207.0098 146.9837 275.0457 235.0089 185.1625	174.9783 163.2186	Chlorogenic acid
	23	19.19	254,278	449.1190	207.0091 234.9587 243.0011 177.0516 285.0846 377.4037	299.0534 353.0652 383.0690 339.0555 395.0833	8-*C*-glucosyl luteolin (orientin)
	24	19.79	254,278	449.0907	431.0489 301.1575 206.9786 215.0161	299.0532, 353.0659, 395.0849, 463.4953 329.0699, 383.0960	6-*C*-glucosyl luteolin (isoorientin)
	25	20.86	254,278	433.1147	206.9915 234.0091 174.9798 251.1581 279.0238	177.7364 245.2327 100.8017	8-*C*-glucosyl apigenin (vitexin)
	6	22.32	254,278	433.1255	175.0077 313.0549 455.0990	168.5285	6-*C*-glucosyl apigenin (isoviterxin)
	5	25.95	254,278	493.1481	206.9978 371.0754 159.0127 351.1284	331.0757	*O*-hexosyl tricin
	4	26.75	254,278	639.1185	191.0127 207.0049 235.0432 253.1692 460.8544	331.0811 315.0479	*O*-hexosyl-*O*- deoxyhexosyl tricin
Branch	21	8.57	254,278	346.0656	206.9941 234.9992 174.9993 191.0522	248.2391	Not identified
	1	16.93	254, 278	595.1387	579.1455 371.1160 249.1300 311.0476 235.0080 207.0031	457.1221 325.0685 427.1048 379.0890 295.0745	Di-*C*,*C*-hexosyl apigenin
Outer culm (5 m)	21	8.65	254, 278	346.0495	206.9909 235.0080 158.9887 174.9798 193.0579	152.0649 257.2977 172.7825	Not identified
	1	16.90	254, 278	595.1733	579.2583 457.1415 371.0852 249.1032 311.0364 206.9530	379.0866 457.1184 427.1094 325.0682	Di-*C*,*C*-hexosyl apigenin
	4	26.62	254, 278	639.1777	557.1886 441.1388 355.1725 175.0428 159.0067	331.0788 315.0678 270.0594 285.0364	*O*-hexosyl-*O*-deoxyhexosyl tricin
Outer culm (1 m)	21	8.59	254, 278	346.0550	207.0078 174.9546 235.0164 218.0567	152.0448 202.7962	Not identified
	1	16.89	254, 278	595.1143	579.1475 249.1167 371.1295 207.0176 175.0050	427.0888 379.0915 295.0598	Di-*C*,*C*-hexosyl apigenin
	26	25.86	254, 278	295.0849	207.0089 219.0139 174.9589 147.0010	135.8380 178.2954	Not identified
Inner culm (5 m)	27	16.95	254, 278	165.0803	146.9779	-	*p*-courmaric acid
Inner culm (1 m)	27	16.88	254, 278	165.0865	146.9779	-	*p*-courmaric acid
Knot (5 m)	27	17.06	254, 278	165.0806	146.9779	-	*p*-courmaric acid
Knot (1 m)	27	16.89	254, 278	165.2503	147.0356	-	*p*-courmaric acid
Rhizome	28	7.05	254,278	330.0130	206.9530 234.9505 174.9735 266.0403	221.4443 259.8027 104.8472	Not identified
	21	8.66	254,278	346.0354	206.9626 233.9144 174.9779 214.9463	152.0740 174.0595	Not identified
	27	16.92	254,278	165.3803	146.9779 159.0007	132.8580	*p*-courmaric acid
Root	27	16.91	254,278	165.0853	146.9894 159.0127	-	*p*-courmaric acid

* Compounds that show pseudomolecular ions in mass spectra in both positive and negative ion modes were listed here and indicated in corresponding chromatograms in Figure S2.

Immunoglobulin E (IgE) is well known as a trigger of allergic reactions [21]. Here, the level of IgE production in Peripheral Blood Lymphocytes (PBL) was used to evaluate anti-allergy activity of the extracts from different parts of bamboo. 

Table 4 shows the anti-allergy activity of the ethanol extracts and the hot water extracts of *P. pubescens*. Compared with the IgE concentration of controls, hot water extracts of leaves, outer culm (5m, 1m), inner culm (5 m), knots (5 m), and rhizomes significantly inhibited the production of IgE in PBL. Among these extracts, leaves showed the strongest anti-allergy activity with an inhibition rate of 42.8%. The inhibition rates of other extracts were 29.3% (outer culm at 5 m), 35.9% (outer culm at 1 m), 35.9% (inner culm at 5 m), 35.9% (knots at 5 m) and 37.2% (rhizomes), respectively. On the other hand, ethanol extracts showed no effect on IgE production in PBL. 

### 2.5. Phytochemical Profile

The chromatographic profiles of each extracts were determined through LCMS analysis (Figures S1 and S2). Very different chromatograms can be seen for the extracts from the leaf, branch, outer culm, inner culm, knot rhizome or root parts, suggesting the ethanol extracts and water extracts from different parts of bamboo have very different chemical compositions. Based on the data from both positive and negative MS and MS/MS spectra, the component of each extract was partially identified referring to the standards or the literature [22,23]. For example, di-*C*,*C*-hexosylapigenin (compound **1**) was first identified in the ethanol extract of outer culm (1 m) for the presence of a pseudomolecular ion at *m/z* 595 [M+H]^+^ and four typical fragment ions of di-*C*, *C*-hexosyl-flavones [22,24]. They are m/z 325 [(M+H)-120-150]^+^, m/z 427 [(M+H)-150-18]^+^, m/z 457 [(M+H)-120-18]^+^ and m/z 379 [(M+H)-120-96]^+^ (Figure S3A). In other extracts, di-*C*,*C*-hexosyl apigenin was identified through the pseudomolecular ion, the typical fragment ions and the retention time referring to that in the ethanol extract of outer culm (1 m). Similarly, *O*-hexosyl-*O*-deoxyhexosyl tricin (compound **4**) was tentatively identified because the presence of a pseudomolecular ion at *m/z* 639 [M+H]^+^, the characteristic fragment ion for *O*-hexosyl-*O*-deoxyhexosyl derivatives at *m/z* 331 [(M+H)-162-146]^+^ [18], fragment ion at *m/z* 561 [(M+H)-60-18]^+^ and 357 [(M+H)-120-162]^+^ (Figure S3B). 6-*C*-Glucosylapigenin (compound **6**) was mainly identified based on the appearance of pseudomolecular ion at *m/z* 433 [M+H]^+^ and typical mono-*C*-glycoside fragment ions at *m/z* 313 [(M+H)-120]^+^, *m/z* 283 [(M+H)-150]^+^ and *m/z* 337 [(M+H)-60-18-18]^+^. The position of the mono-*C*-glycosylation was indicated by the appearance of fragment at *m/z* 341 [(M-H)-90]^−^ and *m/z* 323 [(M-H)-90-18]^−^ [23,25] (Figure S3C). Chlorogenic acid (compound **22**) and *p*-courmaric acid (compound **27**) were identified by their identical retention times, pseudomolecular ions and fragment ions as the corresponding standard compounds. Chlorogenic acid showed a clear psudomolecular at *m/z* 353 [M-H]^−^ and a dominant fragment ion at *m/z* 191 [(M-H)-162]^−^, while *p*-courmaric acid showed a clear psudomolecular at *m/z* 163 [M-H]^−^. Because the complex composition of the extracts, only the fractions showing pseudomolecular ions in both positive and negative ion modes were listed in the table and tentatively identified (Table 5 and Table 6). These fractions were indicated in corresponding chromatograms (Figure S1 and S2), functioning as the markers in the characteristic chromatogram of each extract. 

The results showed that the glycoside, di-*C*,*C*-hexosylapigenin, which existed in the ethanol extracts of leaf, outer culm, inner culm, root and water extracts of leaf and branch (Table 5 and Table 6), is the most common compound in the different parts of bamboo. Besides di-*C*,*C*-hexosylapigenin, three other apigenin derivatives, 6-*C*-glucosylapigenin (compound **6**), 8-*C*-glucosylapigenin (compound **25**) and di-*C*-glycosylapigenin (compound **20**) were also found in different extracts. 6-*C*-Glucosylapigenin was found in the ethanol extract of branch and water extract of leaf, while 8-*C*-glucosylapigenin was only found in the water extract of leaf and di-*C*-glycosylapigenin was found in the ethanol extract of root. Another major component found in these extracts was tricin derivatives. *O*-Hexosyl-*O*-deoxyhexosyl tricin (compound **4**) was found in both ethanol extract and water extract of leaf and outer culm, while *O*-hexosyltricin (compound **5**) was found in the ethanol extracts of leaf and root and the water extract of leaf. Two luteolin derivatives, 6-*C*-glucosylluteolin (compound **24**) and 8-*C*-glucosylluteolin (compound **23**) were also found in the water extract of leaf. In the water extract of outer culm, inner culm, rhizome and root, *p*-courmaric acid (compound **27**) was found. 

Although the components of each extract were only partially identified and a quantitative analysis was not done, we tried to find some hints indicating possible active compounds by comparing the results from the LC-MS and activity assays. Apigenin is a naturally occurring flavonoid, which has been reported to possess various activities, including antioxidation [26], antimutagenic [27], anti-inflammation [28], and anticarcinogenic activities [29], and so on. Its derivatives 6-*C*-glucosyl- apigenin (isovitexin, compound **6**) and 8-*C*-glucosylapigenin (vitexin, compound **25**) were found to have anti-diabetic complication activity and anti-Alzheimer’s disease activity [30]. Here, 8-*C*-glucosylapigenin (compound **25**) was only found in the water extract of leaf that showed the strongest anti-allergy activity among all extracts (Table 4), suggesting 8-*C*-glucosylapigenin had the higher possibility than other three apigenin derivatives to be responsible for the anti-allergy activity. In addition, 8-*C*-glucosylluteolin (orientin, compound **23**), 6-*C*-glucosylluteolin (isoorientin, compound **24**) and chlorogenic acid (compound **22**) were also only found in the water extract of leaf (Table 6). Luteolin and luteolin 7-glucoside had been reported to show allergy-preventive activity [31,32]. Chlorogenic acid had a series of biological effects [33] and also had been found to have allergy-preventive activity [34]. Therefore, the most probable compounds responsible for the anti-allergy of bamboo were 8-*C*-glucosylapigenin, the luteolin derivatives and chlorogenic acid (compound **22**). *O*-Hexosyl-*O*-deoxyhexosyl tricin (compound **4**) mainly appeared in the ethanol extract of outer culm that showed strongest antibacterial and melanin inhibition activity (Table 3 and Table 1), suggesting *O*-hexosyl-*O*-deoxyhexosyl tricin was possibly the compound responsible for the antibacterial and melanin inhibition activity, although tricin had no activity against *S. aureus* [35]. The ethanol extract of inner culm and branch showed best antioxidant activity (Table 2), but we couldn’t identify more compounds from them so far except for 6-*C*-glucosylapigenin (compound **6**) and di-*C*,*C*-hexosyl apigenin (compound **1**). Apigenin was already known as an antioxidant [36,37]. Further studies are needed to find out the exact active compounds responsible for these bioactivities of bamboo.

## 3. Experimental

### 3.1. Plant Materials

Whole plants of 1 or 2-year old *P. pubescens* were harvested at Kurume, Fukuoka Prefecture, Japan. The average height of the harvested bamboo was 14 m. Then, plants were separated into the following parts: leaves, branches, outer culm, inner culm, knots, rhizomes and roots (Figure 1). At that time, the outer culm, inner culm and knots were obtained separately from heights of 5.0 ± 0.3 and 1.0 ± 0.3 m above ground level. Each part was freeze-dried and milled into powder.

Milled freeze-dried *P. pubescens* samples were extracted with 99.5% ethanol at room temperature with a shaker at 200 rpm for 48 h and then filtered. The ethanol extracts were concentrated by a rotary evaporator. The yields of ethanol extracts against each dried powder were as follows: leaves, 4.84%; branches, 1.08%; outer culm (5 m), 4.56%; outer culm (1 m), 4.69%; inner culm (5 m), 0.27%; inner culm (1 m), 0.32%; knots (5 m), 1.47%; knots (1 m), 1.55%; rhizomes, 0.45% and roots, 2.63%. To prepare the hot water extracts, *P. pubescens* samples were extracted with hot water at 120°C for 20 min and the extracted solutions were freeze dried. The yields of hot water extracts were as follows: leaves, 10.4%; branches, 2.67%; outer culm (5 m), 2.96%; outer culm (1 m), 3.69%; inner culm (5 m), 1.60%; inner culm (1 m), 2.21%; knots (5 m), 3.64%; knots (1 m), 4.94%; rhizomes, 2.49% and roots 3.25%.

### 3.2. Melanin Biosynthesis Assay

This assay was performed as previously described by Arung *et al*. [38]. The B16 melanoma cells were maintained in EMEM supplemented with 10% (v/v) fetal bovine serum (FBS) and 0.09 mg/mL theophylline. Cells were incubated at 37 °C in a humidified atmosphere of 5% CO_2_. Cells were placed into a 24-well plate at a density of 1 × 10^5^ cells/mL and incubated for 24 h in medium prior to treatment with extract. After 24 h, the medium was replaced with 998 μL of fresh medium, and 2 μL of ethanol extract dissolved in dimethylsulfoxide (DMSO) or hot water extract dissolved in sterilized water was added. The cells were incubated for an additional 48 h; then the medium was replaced with fresh medium and extract was added again. After 24 h, the remaining adherent cells were used to determine the melanin content and cell viability (see below). To find possible candidates for whitening or tanning agents, we classified the tested extracts into three types (Type A, B, and C). Samples which showed a percentage of melanin content equal to or lower than 20% of cell viability (e.g., CV-MC ≥ 20) were judged as possible whitening agents, and classified as type A. In the other hand, samples which showed a percentage of melanin content equal to or higher than 20% of cell viability (e.g., MC-CV ≥ 20) were judged as possible tanning agents, and classified as type B. Finally, samples showed a percentage of cell viability equal to or lower than 90% were judged to be cytotoxic and classified as type C.

#### 3.2.1. Cell Viability

Cell viability (CV) was determined by use of the microculture tetrazolium technique (MTT) [38]. Culture was initiated, and after incubation, 50 μL of MTT [3-(4,5-dimethylthiazol-2-yl)-2,5-diphenyltetrazolium bromide] in phosphate buffered saline (5 mg/mL) was added to each well. The plates were incubated for 4 h. After removing the medium, formazan crystals were dissolved in 1.0 mL of 0.04 M HCl in isopropanol and the absorbance was measured at 570 nm relative to 630 nm.

#### 3.2.2. Determination of Melanin Content

The melanin content (MC) of cells after treatment with the extract was determined as follows. After removing the medium and washing the cells, the cell pellet was dissolved in 1.0 mL of 1 M NaOH. The crude cell extracts were assayed using a microplate reader (Bio-Tek, Winooski, VT, USA) at 405 nm to determine the melanin content. The results from the samples were analyzed as a percentage of the control culture. Arbutin was used as a positive control.

### 3.3. Antioxidant Assays

#### 3.3.1. Oxygen Radical Absorbance Capacity Assay

The oxygen radical absorbance capacity (ORAC) assay was performed as described previously by Prior *et al.* [39]. Data are expressed as milligrams of Trolox equivalent (TE) per milligram of sample extract (mg TE/mg).

#### 3.3.2. Superoxide Dismutase-Like Activity

Superoxide dismutase (SOD)-like activity was evaluated using the SOD Assay Kit-WST (Dojindo Molecular Technologies, Kumamoto, Japan) according to the method described in previous studies [40]. Sample were dissolved in water or ethanol and added to the WST working solutions (200 μL) containing 2-(4-iodophenyl)-3-(4-nitrophenyl)-5-(2, 4-disulfophenyl)-2-*H*-tetrazolium in 50 mM carbonate buffer (pH 10.2). An enzyme working solution (20 μL) containing xanthine oxidase in the same buffer was added and then incubated for 10 min. The absorbance of each sample was measured at 450 nm in a Tecan Spectra microplate reader (Tecan Japan, Kanagawa, Japan). One unit of SOD-like activity was defined as the amount of extract in 20 μL of sample solution that inhibits the reduction reaction of WST-1 with superoxide anions by 50%. The SOD-like activity (U/mg) of each extract was calculated using the 50% inhibition value (IC_50_) of the extract.

#### 3.3.3 ABTS Radical Cation Decolorization Assay

The ABTS assay was mostly based on the methods described by Re *et al.* [41] in which ABTS^•+^, the oxidant, was generated by persulfate oxidation of ABTS [2,2'-azinobis (3-ethylbenzothiazoline-6-sulfonic acid)]. Specifically, to 5 mL of 7 mM ABTS ammonium aqueous solution, 88 µL of 140 mM potassium peroxydisulfate (K_2_S_2_O_8_) was added, and the resulting mixture was then allowed to stand at room temperature for 12-16 h, yielding a dark blue solution. The mixture was then adjusted by 99.5% ethanol so that it gave an absorbance of 0.7 ± 0.02 units at 734 nm (UVmini-1240, Shimadzu, Kyoto, Japan) to make the working solution. One milliliter of working solution was mixed with 10 µL of extract dissolved in ethanol and shaken well for 10 s; after 4 min of incubation at 30 °C, the absorbance of the reaction mixture was measured at 734 nm.

### 3.4. Antibacterial Assay

The antibacterial assay was mostly based on the methods described by Tanaka *et al.* [10]. *S. aureus* (NBRC 1273) was used for the antibacterial assay. A single colony of the test strain was taken and 5 mL of nutrient broth medium was added to it. This culture was incubated at 37 °C ± 1 °C, 120 rpm for 20 h. It was then added to the bacterial suspension to prepare a bacterial concentration at 10^5^ CFU/mL. The bacterial solution was used for the subsequent antibacterial assay. Each sample was dissolved in DMSO for ethanol extract or sterilized water for hot water extract at maximum concentration. Into each well of a 96-well plate were added 133.5 µL of NB medium, 15 µL of bacteria suspension, and 1.5 µL of solvent with or without each sample. Also, sorbic acid was used as a positive control. The plate was incubated at 37 °C ± 1 °C, 1160 rpm for 18 h. Finally, bacterial growth was measured by a microplate reader at 630 nm (Biotek-ELX800, BioTek). The minimum inhibitory concentration (MIC) is the lowest concentration of an antibacterial agent required to completely inhibit the growth of a particular bacteria, while the minimum bactericidal concentration (MBC) is the lowest concentration of an antibacterial agent required to kill the bacteria. Here, the MIC of active extracts was determined through the antibacterial assay using gradient concentrations. And MBC of them were further determined as follows: a 20 μL aliquot was taken from the wells that treated with extract at higher concentration than its MIC and mixed with 180 μL of fresh medium. Then, 100 μL of the mixture was used to do the subculture on nutrient agar plate. After 24 h incubation at 37 °C ± 1 °C, the colony formation was evaluated. The minimum concentration that leaded to no colony growing on the agar plate was considered as the MBC. 

### 3.5. Immunoglobulin E (IgE) Production Assay

Peripheral blood lymphocytes (PBL) were first separated from heparinized blood of healthy donors using Ficoll-Paque Plus (GE Healthcare, Uppsala, Sweden). And then, PBL cells were cultured in ERDF medium (Kyokuto Pharmaceuticals, Tokyo, Japan) supplemented with 5% FBS, 10% human plasma, 10 ng/mL of recombinant human IL-4 and IL-6 (R&D Systems, USA), 10 μg/mL of muramyl dipeptide (MDP) (Sigma, St.Louis, MO, USA) and 100 ng/mL of the cedar pollen antigen Cry j 1 (Hayashibara Biochemical Laboratories, Okayama, Japan) at the density of 2.0 × 10^6^ cells/mL. 198 μL of such cell suspension and 2 μL of 6mg/ml extract in 10% DMSO solution were added into 96-well plates (final concentration of extract was 60μg/mL). The plate was incubated in a humidified 37°C, 5% CO_2_ incubator for 10 days. The total IgE concentration in the supernatant was measured by sandwich ELISA (enzyme-linked immunosorbent assay). Briefly, 96-well microplates were coated with anti-human IgE antibody (Biosource, Camarillo, CA, USA). The antibody-coated wells were blocked with 1.0% BSA, following by adding the samples. After washing with PBS containing 0.05% of Tween 20 for three times, biotin-conjugated antihuman IgE antibody (Biosource) and horseradish peroxidase-conjugated streptavidin were added. Finally, a substrate solution [0.1 M citrate buffer (pH 4.0) containing 0.003% of H_2_O_2_ and 0.3 mg/mL *p*-2,2'-azino-bis (3-ethylbenzothiazoline-6-sulfonic acid) diammonium salt] was added. After 15 min, the absorbance was measured at 414 and 490 nm by the microplate reader (iMark, Bio-Rad, Hercules, CA, USA). The relative IgE production was calculated according to the absorbance at 414 nm and 490 nm, and the final inhibition rate was calculated using the following formula: Inhibition rate (%) = (1 − IgE production in treated cells/IgE production in control cells) × 100.

### 3.6. LCMS Analysis

All extracts were subjected to LCMS analysis using a high-speed liquid chromatography mass spectrometry that combines with iron-trap and time-of-flight technologies (LCMS-IT-TOF, Shimadzu, Tokyo, Japan). The instrument was fitted with an Inertsil ODS-3, 5μm, 1.5 × 150 mm column (GL Science, Tokyo, Japan). The oven temperature was set at 40 °C. A mobile phase composed of solvent A (0.3% acetic acid in water) and B (0.3% acetic acid in acetonitrile or methanol) was employed for the separation. Acetonitrile was used in solvent B for the analysis of ethanol extract, while methanol was used for water extract. The mobile phase was consecutively programmed as follows: 0~60 min, A 90~0%, B 10%~100%; 60~65 min, A 0, B 100%; 65~66 min, A 0%~90%, B 100%~10%; a 10 min post-run was used after each analysis. The total flow rate was 0.15 mL/min. Basing on the previous result of HPLC-PDA analysis, the LC chromatograms of ethanol extracts and water extracts were obtained at UV 254 nm, 326 nm and 254, 278 nm, respectively. The MS instrument was operated using an ESI source in both positive and negative ionization mode with survey scans acquired from *m/z* 100 to 1000 for both MS and MS/MS. Ionization parameters were as follows: probe voltage, ±4.5 kV; nebulizer gas flow, 1.5 L/min; CDL temperature, 200 °C; heat block temperature, 200 °C. 

The samples were dissolved with initial mobile phase (1 mg/mL) and filtered through a 0.45-μm filter. A volume of 5 μL of each sample was injected for the analysis. 8 compounds that had been found in different bamboo species were analyzed and used as a standard. They were catechin (Sigma-Aldrich, Munich, Germany), caffeic acid (Tokyo Chemical Industry, Tokyo, Japan), syringic acid (Tokyo Chemical Industry), chlorogenic acid (Sigma-Aldrich), *p*-courmaric acid (Sigma-Aldrich), rutin (Wako, Tokyo, Japan), *trans*-ferulic acid (Tokyo Chemical Industry) and luteolin-7-*O*-glucoside (EXTRASYNTHESE, Genay, France). 

## 4. Conclusions

In this study, the effect of ethanol and hot water extracts of various parts of bamboo on the melanin biosynthesis regulation (inhibition or stimulation), antioxidation, antibacterial and anti-allergy were comparatively evaluated. We found that the extracts showed different bioactivities in different degrees. For the melanin biosynthesis inhibition, the hot water extracts of outer culm (5 m) and rhizome showed the best activities. For the melanin biosynthesis stimulation, the ethanol extract of inner culm (1 m) showed the strongest activity. For the antioxidant activity, the ethanol extracts of inner culm (1 m), branch and inner culm (5 m) showed the strongest activities. For antibacterial activity against *S. aureus*, the ethanol extracts of outer culm (5 m and 1 m) showed the strongest activities. The MIC and MBC for both extracts were 400 and 1600 μg/mL, respectively. For anti-allergy activity, the water extract of leaf showed the best IgE inhibition effect. Extracts from the outer culm and inner culm were found to be the most active extracts. 

Different parts of bamboo showed different bioactivities, which also varied with the extraction solvent. The difference in chromatographic profile and identified component to some extent explained the different bioactivities of these extracts. The most possible compounds responsible for anti-allergy activity of this bamboo were 8-*C*-glucosyl apigenin, luteolin derivatives and chlorogenic acid. *O*-hexosyl-*O*-deoxyhexosyl tricin was the possible compound responsible for the antibacterial and melanin inhibition activity of bamboo, while apigenin derivatives might be the compounds responsible for the antioxidant activity. This information would be helpful for the further research on the active compounds in bamboo. Taken together, our study provides valuable data to support that bamboo has great potential to be used in the cosmetic industry as well as other health-related industry. 

## Supplementary Materials

Supplementary materials can be accessed at: http://www.mdpi.com/1420-3049/19/6/8238/s1.
