# Supplementary material for: Biological Activities and Phytochemical Profiles of Extracts from Different Parts of Bamboo (Phyllostachys pubescens)
Source: Molecules. 2014 Jun 18;19(6):8238–60. doi: 10.3390/molecules19068238 (PMC6270728; doi:10.3390/molecules19068238)

Supplementary Information

Figure S1. LCMS-IT-TOF chromatogram of ethanol extracts from various parts of bamboo. The ethanol extracts from the leaf, branch, out culm (5 m), out culm (1 m), inner culm (5 m), inner culm (1 m), knot (5 m), knot (1 m), rhizome and root of bamboo were analyzed by LCMS-IT-TOF (Shimadzu, Tokyo, Japan). Chromatograms were obtained under the following conditions: The column was Inertsil ODS-3 (5µm, 1.5 × 150 mm, GL Science, Tokyo, Japan). The oven temperature was set at 40 °C. A mobile phase composed of solvent A (0.3% acetic acid in water) and B (0.3% acetic acid in acetonitrile) was employed for the separation. The mobile phase was consecutively programmed as follows: 0~60 min, A 90%~0%, B 10%~100%; 60~65 min, A 0, B 100%; 65~66 min, A 0%~90%, B 100%~10%; a 10 min post-run was used after each analysis. The total flow rate was 0.15 mL/min. Chromatogram obtained at 254 nm was showed here. The peaks listed in Table 5 were indicated in the chromatograms.

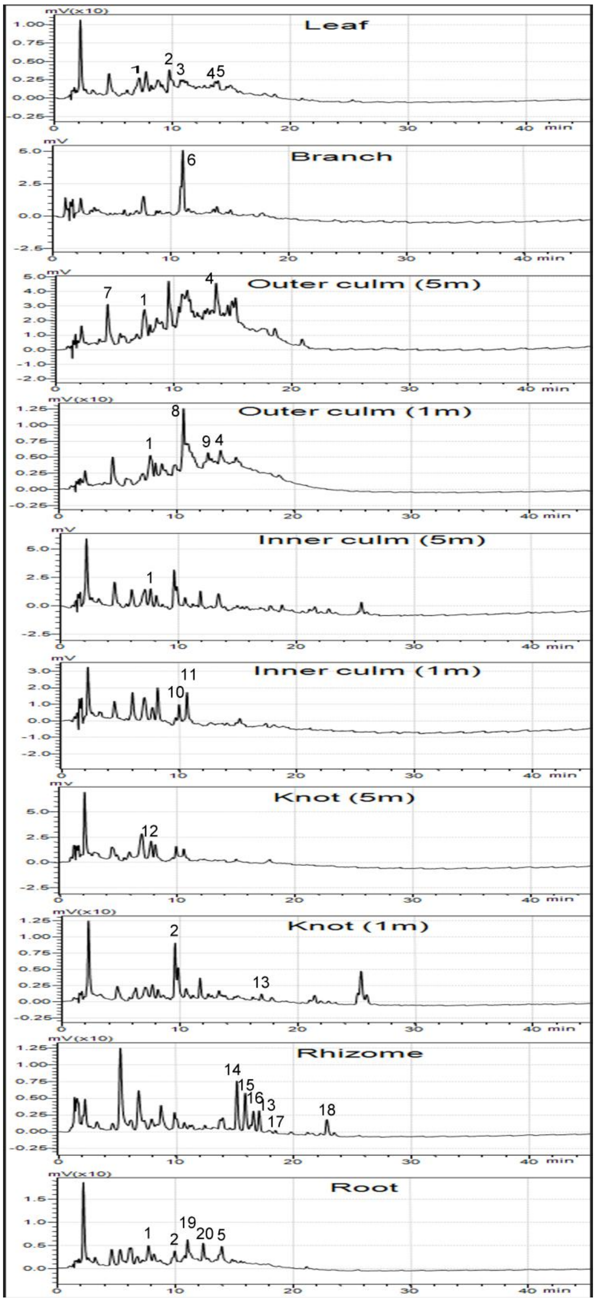

Figure S2. LCMS-IT-TOF chromatogram of water extracts from various parts of bamboo. The water extracts from the leaf, branch, out culm (5 m), out culm (1 m), inner culm (5 m), inner culm (1 m), knot (5 m), knot (1 m), rhizome and root of bamboo were analyzed by LCMS-IT-TOF (Shimadzu, Tokyo, Japan). Chromatograms were obtained under the following conditions: The column was Inertsil ODS-3 (5 µm, 1.5 × 150 mm , GL Science, Tokyo, Japan). The oven temperature was set at 40 ℃. A mobile phase composed of solvent A (0.3% acetic acid in water) and B (0.3% acetic acid in methanol) was employed for the separation. The mobile phase was consecutively programmed as follows: 0~60 min, A 90%~0%, B 10%~100%; 60~65 min, A 0, B 100%; 65~66 min, A 0%~90%, B 100%~10%; a 10 min post-run was used after each analysis. The total flow rate was 0.15 mL/min. Chromatogram obtained at 254 nm was showed here. The peaks listed in Table 6 were indicated in the chromatograms.

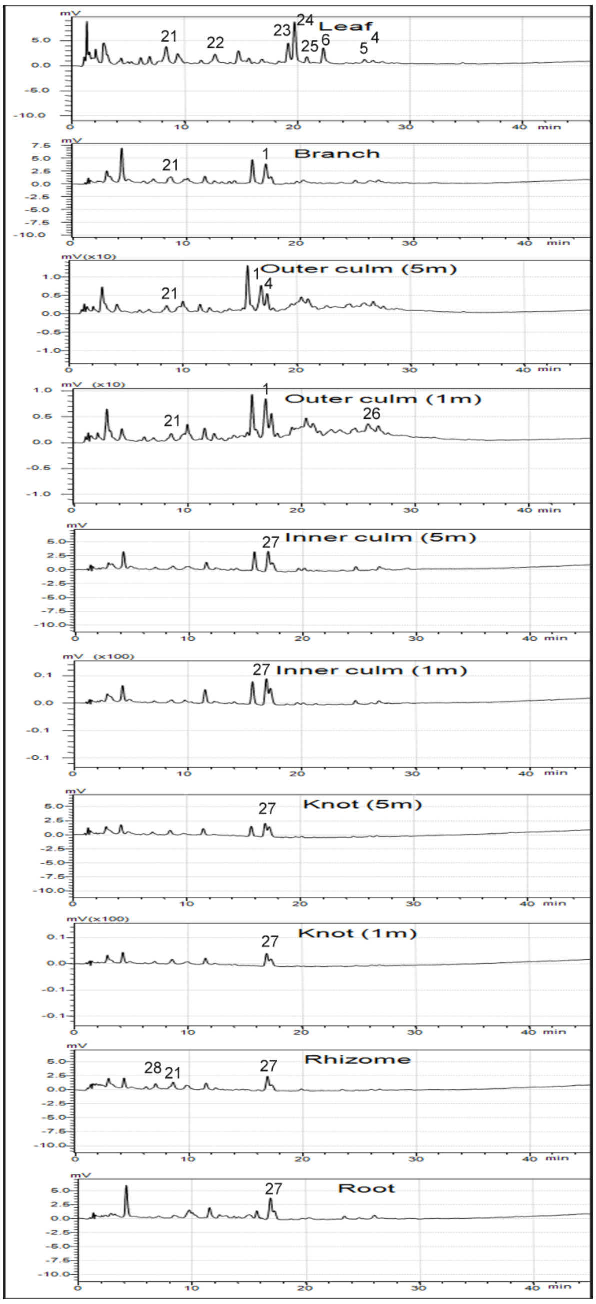

Figure S3. Fragmentation patterns of three flavonoid compounds from bamboo—examples of compound identification by fragment ions. (**A**) Di-*C*, *C*-hexosyl apigenin (compound 1) was identified basing on the appearance of pseudomolecular ion [M+H]$^+$ at *m/z* 595, fragment ions [(M+H)-120-150]$^+$ at *m/z* 325, [(M+H)-150-18]$^+$ at *m/z* 427, [(M+H)-120-18]$^+$ at *m/z* 457, [(M+H)-120-60-18-18]$^+$ at *m/z* 379 and [(M+H)-123]$^+$ at 472. (**B**) *O*-hexosyl-*O*-deoxyhexosyl tricin (compound 4) was tentatively identified because the presence of a pseudomolecular ion at *m/z* 639 [M+H]$^+$, the characteristic fragment ion for *O*-hexosyl-*O*-deoxyhexosyl derivatives at *m/z* 331 [(M+H)-162-146]$^+$, fragment ion at *m/z* 561 [(M+H)-60-18]$^+$ and 357 [(M+H)-120-162]$^+$. (**C**) 6-*C*-glucosyl apigenin (compound 6) was mainly identified basing on the appearance of pseudomolecular ion at *m/z* 433 [M+H]$^+$ and typical mono-*C*-glycoside fragment ions at *m/z* 313 [(M+H)-120]$^+$, *m/z* 283[(M+H)-150]$^+$ and *m/z* 337 [(M+H)-60-18-18]$^+$. The mono-*C*-glycosylation was indicated at position 6 because of the dominant fragment at *m/z* 341 [(M-H)-90]$^-$ and *m/z* 323 [(M-H)-90-18]$^-$.

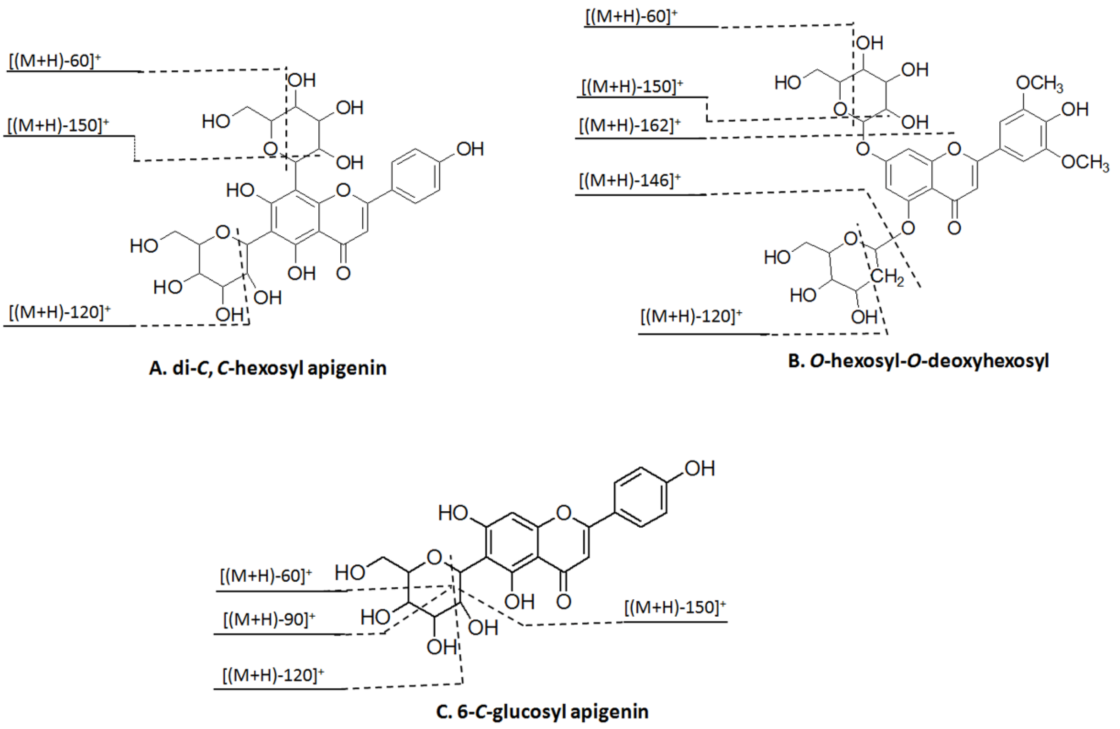

Supplement: Supplementary file 1 [file molecules-19-08238-s001.pdf]
